# Biosynthesis of (*R*)-(-)-1-Octen-3-ol in Recombinant *Saccharomyces cerevisiae* with Lipoxygenase-1 and Hydroperoxide Lyase Genes from *Tricholoma matsutake*

**DOI:** 10.4014/jmb.2001.01049

**Published:** 2020-02-17

**Authors:** Nan-Yeong Lee, Doo-Ho Choi, Mi-Gyeong Kim, Min-Ji Jeong, Hae-Jun Kwon, Dong-Hyun Kim, Young-Guk Kim, Eric di Luccio, Manabu Arioka, Hyeok-Jun Yoon, Jong-Guk Kim

**Affiliations:** 1 School of Life Science and Biotechnology, Kyungpook National University, Daegu 4566, Republic of Korea; 2 School of Life Science, College of Natural Sciences, Kyungpook National University, Daegu 41566, Republic of Korea; 3 Department of Biotechnology, The University of Tokyo 1-1-1 Yayoi, Bunkyo-ku Tokyo 11-8657 Japan

**Keywords:** (*R*)-(-)-1-octen-3-ol, *Tricholoma matsutake*, lipoxygenase, hydroperoxide lyase, *Saccharomyces cerevisiae*

## Abstract

*Tricholoma matsutake* is an ectomycorrhizal fungus, related with the host of *Pinus densiflora*. Most of studies on *T. matsutake* have focused on mycelial growth, genes and genomics, phylogenetics, symbiosis, and immune activity of this strain. *T. matsutake* is known for its unique fragrance in Eastern Asia. The most major component of its scent is (*R*)-(-)-1-octen-3-ol and is biosynthesized from the substrate linoleic acid by the sequential reaction of lipoxygenase and peroxide lyase. Here, we report for the first time the biosynthesis of (*R*)-(-)- 1-octen-3-ol of *T. matsutake* using the yeast *Saccharomyces cerevisiae* as a host. In this study, cDNA genes correlated with these reactions were cloned from *T. matsutake*, and expression studies of theses genes were carried out in the yeast *Saccharomyces cerevisiae*. The product of these genes expression study was carried out with Western blotting. The biosynthesis of (*R*)-(-)- 1-octen-3-ol of *T. matsutake* in recombinant *Saccharomyces cerevisiae* was subsequently identified with GC-MS chromatography analysis. The biosynthesis of (*R*)-(-)-1-octen-3-ol with *S. cerevisiae* represents a significant step forward.

## Introduction

*Tricholoma matsutake*, found in the Korean peninsula, Japan and China, have been regarded as precious food for a long time. Nowadays, it is famous symbol viand in Korea and Japan. *T. matsutake* is high-value food because not only its taste and smell but also its notoriously difficulty of artificial cultivation. The fruiting body of *T. matsutake* is formed by the complex interaction of several ecological factors in specific forest of *Pinus densiflora* [11, 21]. Unfortunately, the occurrence of *T. matsutake* fruiting body in nature is steadily decreasing and the demand for the artificial cultivation of *T. matsutake* is rising. In 1983, Hiroshima Forestry Examination Center had succeeded in the artificial cultivation but failed to preserving this cultivating artifact. Since 1983, lots of studies have been reported and recently the National Forest Research Institute, Korea and Taki Chemical Industry, Japan had succeeded the artificial cultivation of *T. matsutake* in 2010 and 2018, respectively [17, 20]. But most of studies had failed to maintain the cultivation and little is known about its artificial cultivation [23, 31]. Moreover, transplantation of *P. densiflora* trees infected with *T. matsutake* attempted by Japan and Korea had failed to yield the mushroom with a meaningful number [12, 16]. The genomic study of *T. matsutake* was carried out by Dr Min’s research group in January 2020 [28]. Thus, in this study, not artificial cultivation but rather production of the *T. matsutake* flavor is the focus of this study. We expect the outcome of our work to help producing the (*R*)-(-)-1-octen-3-ol at a low price and at a significant yield by cloning the genes encoding the lipoxygenase and hydroperoxide lyase of *T. matsutake* into industrial microorganisms such as *Escherichia coli* and *Saccharomyces cerevisiae*.

**Fig. 1:**
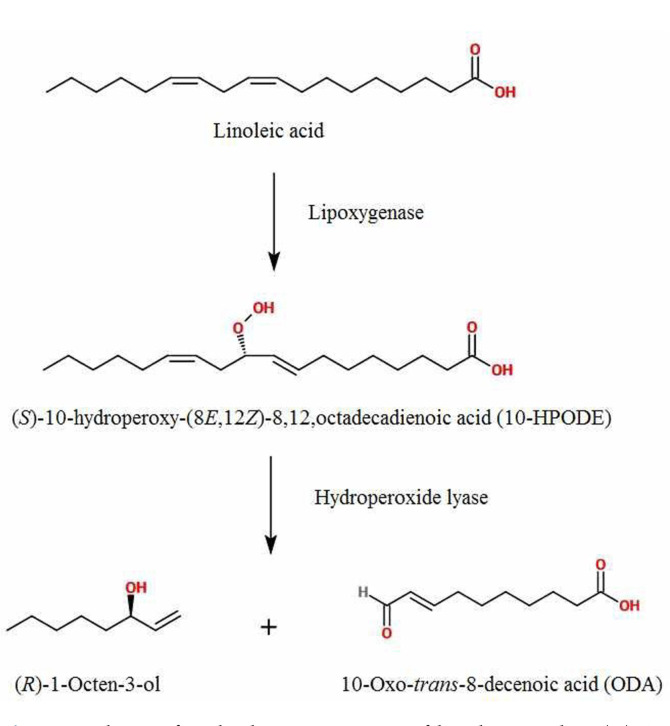
Pathway for the bioconversion of linoleic acid to (*R*)-1- octen-3-ol by lipoxygenase and hydroperoxide lyase. Lipoxygenase participate in transforming linoleic acid into (S)-10- hydroperoxy-(8E,12Z)-8, 12, octadecadienoic acid (10-HPODE) and Hydroperoxide lyase participate in producing (*R*)-1-octen-3-ol.

Among the 9 flavor components, 1-octen-3-ol known as matsutake alcohol comprise of a significant proportion of flavor components formed by *T. matsutake* [4, 8, 30]. As one of the aromatic compound of *T. matsutake*, 1-octen-3-ol participate in protection of the host from harmful insects such as bark beetle and *Proisotoma minuta* that eat fruiting bodies and spores [3, 33]. The racemic 1-octen-3-ol was chemically synthesized and is composed of two enantiomers with equal amounts, and each enantiomer reacts differently depending on the substrates. Like many biological molecules consist of two enantiomers that show opposite effects against each other [1, 10, 14], (*R*)-(-)-1-octen-3-ol has a stronger mushroom-like flavor than the racemic 1-octen-3-ol and (S)-(+)-1-octen-3-ol that has a moldy and grassy smell [5, 19, 27]. Therefore, it is important to isolate the (*R*)-(-)-1-octen-3-ol using the chemically synthesized 1-octen-3-ol in food. Biosynthesis of (*R*)-(-)-1-octen-3-ol can be formed through the aerobic oxidation of linoleic acid using a specific enzymatic reaction [38]. By lipoxygenase treatment, linoleic acid is oxidized into (S)-10-hydroperoxy-(8E,12Z)-8, 12, octadecadienoic acid (10-HPODE) and a polyunsaturated fatty acid. Cleavage into (*R*)-(-)-1-octen-3-ol by hydroperoxide lyase is next (Fig. 1). Unlike other species that includes fungi, plants and mushroom that have lipoxygenase and hydroperoxide lyase, *T. matsutake* produces different final products and yield more (*R*)-(-)-1-octen-3-ol [26, 32, 34]. Although studies on the bioconversion of linoleic acid into (*R*)-(-)-1-octen-3-ol by homogenates of other mushrooms such as *Pleurotus pulmonarius*, *Lentinus decadetes* and *Agaricus bisporus* have been reported, there are only a few studies on the lipoxygenase and hydroperoxide lyase from *T. matsutake* that are insufficient for a commercialization of (*R*)-(-)-1-octen-3-ol [2, 9, 15, 25].

*E. coli* is a gram negative, facultative anaerobic, coliform bacterium that commonly found in the lower intestine of warm-blooded organisms. As harmless yeast, its benefits hosts by producing nutrient and preventing invasion of pathogenic bacteria. Since 1885, E.coli has been used for experiments because it could be grown and cultured easily and inexpensively in a laboratory setting [13, 36]. *S. cerevisiae* has been known as desirable microorganism closely related with humans because of participation in producing the fermented food like beer, wine and bread. The genetic information of *S. cerevisiae* is well known and the yeast can easily be engineered, which has been an attractive microorganism in industrial biotechnology [6, 35]. In addition with transgenic *S. cerevisiae*, successful researches have been reported for the production of high value materials such as fuel and chemical [18, 24, 29]. Therefore, the biosynthesis of (*R*)-(-)-1-octen-3-ol using engineered *S. cerevisiae* to manufacturing the various *T. matsutake* flavor products has a considerable commercial value. In this study, gene sets introduced into *S. cerevisiae* for synthesizing the highest amount of (*R*)-(-)-1-octen-3-ol were identified among lipoxygenase-1, lipoxygenase-2, lipoxygenase-3 and hydroperoxide lyase genes of *T. matsutake*. Furthermore, the optimal biosynthesis conditions of (*R*)-(-)-1-octen-3-ol in transgenic yeast were studied to effectively generate and extract (*R*)-(-)- 1-octen-3-ol.

## Materials and Methods

### Cloning the Genes of Lipoxygenase and Hydroperoxide Lyase from *Tricholoma matsutake*

For producing a yeast expression vectors, the recombinant plasmid was prepared and whole RNA extract from *T. matsutake* fruiting bodies was isolated using liquid nitrogen and TRIzo1 reagent. Then cDNA was synthesized from whole RNA by Accuscript High Fidelity 1^st^ strand cDNA Synthesis Kit (Stratagene, USA). The lipoxygenase and hydroperoxide lyase genes were amplified from the synthesized cDNA by RT-PCR using PrimeSTAR HS Polymerase (TaKaRa, Japan). The primer sequences and restriction enzymes used for each gene are described in Table 1. PCR reaction conditions for each gene were set as follows: pre-denaturation at 98°C for 3 min followed by denaturation at 98°C for 10 sec; annealing and extension of lipoxygenase-1 gene at 60°C for 15 sec and 72°C for 3 min respectively, lipoxygenase-2 gene at 58°C for 15 sec and 72°C for 4 min respectively, lipoxygenase-3 gene at 56°C for 15 sec and 72°C for 4 min respectively, and hydroperoxide lyase gene at 59°C for 5 sec and at 72°C for 2 min respectively, with a total of 30 reaction cycles.

Because the PrimeSTAR HS Polymerase (TaKaRa) has a 3’ to 5’ exonuclease activity, A-tailing of the amplified PCR product was conducted with the TA-cloning Reagent Set for PrimeSTAR. The A-tailed PCR products were cloned into pGEM Easy T-vectors (Promega, USA) and ligated vectors of each gene were transformed into *E. coli* DH5α competent cells (TaKaRa). After the transformation reaction, the *E. coli* were incubated overnight in a Luria Broth (LB) agar containing ampicillin, isopropyl β-D-1-thiogalactopyranoside (IPTG) and X-gal at 37°C. From the incubated *E. coli*, plasmids were extracted by Higene Plasmid Mini Prep Kit (BioFact, Korea). Then agarose gel electrophoresis was conducted to confirm the insertion of the target genes. By using ABI BigDye Terminator v3.1 Cycle Sequencing Kits (Applied Biosystems, USA) and ABI 3730xl DNA Analyzer (Applied Biosystems), DNA sequencing of cloned vectors were conducted.

### Yeast Transformation

With C-terminal peptide that encoding a poly-histidine tag and V5 epitope to detect the recombinant protein, pYES3/CT and pYES2/CT vectors were used for expressing target genes in yeast. The T-vectors inserted with lipoxygenase-1, lipoxygenase-2, lipoxygenase-3 and hydroperoxide lyase genes, which have 100 percentage of homology by comparison with cDNA sequences of *T. matsutake*, were selected by DNA sequencing analysis of the cloned vectors. Using specific restriction enzymes, each gene of the T-vectors was cut for ligation to yeast expression vectors. While ligation, the 3 types of lipoxygenase genes were inserted into pYES3/CT vector and the hydroperoxide lyase gene was inserted into the pYES2/CT vector. The ligated vectors were transformed into *E. coli* and incubated overnight in LB agar with ampicillin at 37°C. After incubation, Higene^TM^ Plasmid Mini Prep Kit (BioFact) was used to extract the plasmids from *E. coli*. The extracted plasmids with inserted target genes were resolved on a agarose electrophoresis gel and sequences were confirmed correct by DNA sequencing (Macrogen, Korea).

Using a S.c EasyComp Transformation Kit (Invitrogen, USA), each cloned vector was transformed into *S. cerevisiae*. The transformed cells were incubated at 30°C for 2~3 days in SC minimal medium (0.67% yeast nitrogen base, 0.192% yeast synthetic drop-out medium supplement, 2% glucose and 2% agar) in separate situation because of the different auxotrophic markers in each expression vector for the selection of yeast transformants: without tryptophan for pYES3/CT transformants and without uracil for pYES2/CT transformants. Each cloned gene was detected by a colony PCR method.

### Expression and Detection of the Recombinant Protein

The pYES3/CT or pYES2/CT vectors have only a GAL1 promoter. Thus, the transcription of each gene could be induced by adding galactose as the carbon source. The transformants were pre-cultivated overnight at 180 rpm in the appropriate SC selectable medium with 2% raffinose as a carbon source at 30°C. Pre-cultivated cells were inoculated into 100 ml of fresh SC induction medium with 2% galactose for carbon source and incubated at 30°C for 8 h at 180 rpm. The induced cells were harvested and disrupted with bead beater that consist of sodium phosphate lysis buffer (50 mM sodium phosphate, 1 mM Phenylmethanesulfonyl fluoride (PMSF), 5% glycerol and 2%Triton X-100; pH 6.5) and acid-washed glass beads (0.4–0.6 mm size). Then, the crude protein supernatant was obtained by centrifugatinn at 4°C for 20 min and the proteins were quantified with a Pierce BCA Protein Assay Kit (Invitrogen).

Using western blot analysis, the recombinant proteins were detected and the protein samples were separated by SDS-PAGE with a 10% polyacrylamide gel at 100 V and transferred to nitrocellulose membranes for 2 h at 50 V. The membranes were blocked with 5% skim milk in 1× TBS buffer containing 0.05%Tween-20 for 90 min at room temperature. To detect the recombinant proteins in membranes, reaction with a 1:5,000 dilution of Anti-V5 Mouse monoclonal antibody (Invitrogen) and a 1:1,000 dilution of glyceraldehyde-3-phosphate dehydrogenase (GAPDH) antibody (Invitrogen) as the loading control antibody was incubated at 4°C overnight. After the membranes were reacted with a 1:50,000 dilution of Rabbit Anti-Mouse polyclonal secondary antibody (Abcam Inc., USA) at room temperature for 90 min. In this process, signals of the target proteins were detected using the Amersham ECL Prime Western Blotting Detection Reagent (GE Healthcare Life Sciences, USA).

### Analysis of (*R*)-(-)-1-Octen-3-ol Production based on Lipoxygenase and Hydroperoxide Lyase Combination by Gas Chromatography–Mass Spectrometry

To determine which gene set is capable for synthesizing the highest amount of (*R*)-(-)-1-octen-3-ol, various combinations of gene encoding proteins were reacted with the substrate, linoleic acid. Before inoculation into 100 ml of fresh SC induction medium and incubation at 30°C for 8 h, transformants introduced lipoxygenase-1, -2, -3and hydroperoxide lyase gene separately were pre-cultivated overnight at 30°C in 30 ml of appropriate SC selectable medium. The crude proteins from the induced cells were extracted using a bead beater with sodium phosphate lysis buffer and quantified by a Pierce BCA Protein Assay Kit (Invitrogen). Mixed proteins suspensions (1 mg/ml) in various combinations were reacted overnight at 4°C with 1.5 mM linoleic acid adding 0.2% Tween-20 in total volume of 3 ml. Then, the volatile flavor components were extracted by solid phase micro-extraction (SPME) as follow: samples were transferred into the 20 ml headspace vial and the samples with vial were equilibrated at 70°C for 5 min, followed by adsorption onto the fiber were analyzed by Gas chromatography-mass spectrometry (Aqilent 789OB GC & 5977B MSD) with the DB-WAX column (60 m × 250 μm × 0.25 μm) and helium carrier gas (flow rate of 1 mL/min). The column temperature was programmed as follow: initial oven temperature of 40°C increased to 120°C at 2°C/min and from 120°C to 240°C at 20°C/min. Then the temperature was set at 240°C for 5 min. The injector temperature was 250°C and total running time was 53 min. The amount of (*R*)-(-)-1-octen-3-ol synthesized by recombinant enzyme activity was analyzed compared to the standard 1-octen-3-ol (Sigma, USA).

### Co-Transformation and Protein Expression

In order to biosynthesize the (*R*)-(-)-1-octen-3-ol in yeast, the lipoxygenase-1 and hydroperoxide lyase genes were introduced into *S. cerevisiae*. The expression vectors lipoxygenase-1/pYES3 and hydroperoxide lyase/pYES2 were mixed in a 1:1 ratio and transformed into *S. cerevisiae* by S.c EasyComp Transformation Kit (Invitrogen). The co-transformants were spread on SC minimal medium without tryptophan and uracil, and incubated at 30°C for 2~3 days. After incubation, the presence of the two genes from the co-transformants was determined by colony PCR.

In order to determine the expression level of the lipoxygenase-1 and hydroperoxide lyase proteins based on incubation time, co-transformants were pre-cultivated overnight at 30°C in SC selectable medium without tryptophan and uracil. The pre-cultivated co-transformants were incubated in fresh SC induction medium without tryptophan and uracil at 30°C. Then induced cell isolates were collected separately at 0, 8, 16, 28 and 32 h in incubation. After crude proteins were extracted by bead beater with sodium phosphate lysis buffer from the induced cells, they were quantified with a Pierce BCA Protein Assay Kit (Invitrogen). Quantified samples were separated by SDS-PAGE in a 4~20% gradient polyacrylamide gel at 100 V and transferred to nitrocellulose membranes for 2 h at 50 V. The membranes were blocked with 5%skim milk in 1× TBS buffer containing 0.05% Tween-20 for 90 min at room temperature. To detect the recombinant proteins on the membranes, reaction with a 1:5,000 dilution of Anti-V5 Mouse monoclonal antibody (Invitrogen) and a 1:1,000 dilution of GAPDH antibody (Invitrogen) as the loading control antibody was incubated at 4°C overnight. Next, the membranes were reacted with a 1:50,000 dilution of Rabbit Anti-Mouse polyclonal secondary antibody (Abcam Inc., USA) at room temperature for 90 min. In this process, the signals of the target proteins were detected by using the Amersham ECL Prime Western Blotting Detection Reagent (GE Healthcare Life Sciences).

In order to identify the (*R*)-(-)-1-octen-3-ol biosynthesis from the yeast with lipoxygense-1 and hydroperoxide lyase genes, the co-transformants were incubated in the presence of the linoleic acid, as a substrate. The co-transformants used for identifying the (*R*)-(-)-1-octen-3-ol biosynthesis were pre-cultivated overnight in SC selectable medium without tryptophan and uracil at 30°C. After overnight cultivation, the cells were inoculated into 100 ml of SC induction medium containing 1.5 mM linoleic acid and 0.2%Tween-20, followed by an incubation at 30°C for 20 h. Next, the co-transformants in the presence of the linoleic acid, cells and medium were separated by centrifugation at 1,500 rpm for 10 min. The harvested cells were disrupted by the bead beater with sodium phosphate lysis buffer (50 mM sodium phosphate, 1 mM PMSF, 5% glycerol and 2% Triton X-100; pH 6.5) and acid-washed glass beads (0.4–0.6 mm size). The cell lysates were then collected by centrifugation at 4°C for 20 min. The volatile flavor components were then extracted from cell lysates by SPME as follow: samples were transferred into the 20 ml headspace vial and the samples with vial were equilibrated at 70°C for 5 min, followed by adsorption onto the fiber (CAR/DVB/PDMS/Gray) for 30 min. The absorbed volatile flavor components were analyzed by GC-MS (Agilent 789OB Gas Chromatograph & 5977B MSD mass spectrometry) with the DB-WAX column (60 m × 250 μm × 0.25 μm) and helium carrier gas (flow rate of 1 ml/min). The column temperature was programmed as follow: initial oven temperature of 40°C increased to 120°C at 2°C/min and from 120°C to 240°C at 20°C/min. Then the temperature was set at 240°C for 5 min. The injector temperature was set to 250°C for a total running time of 53 min.

### Analysis of Optimal Reaction Condition for (*R*)-(-)-1-octen-3-ol Biosynthesis

In order to optimize the (*R*)-(-)-1-octen-3-ol biosynthesis in yeast with the lipoxygense-1 and hydroperoxide lyase genes, the co-transformants were incubated under different conditions. The co-transformants for incubation were pre-cultivated overnight in SC selectable medium without tryptophan and uracil at 30°C. Then, pre-cultured cells were collected by centrifugation at 1,500rpm for 10 min and suspended to an optical density at 600 nm (OD_600_) of 0.8 in 100 ml.

To analyze (*R*)-(-)-1-octen-3-ol biosynthesis based on linoleic acid concentrations, the overnight cultivated cells were inoculated into 100 ml of SC induction medium with appropriate concentrations of linoleic acid (0~0.1 M) and 0.2% Tween-20. After incubation at 30°C for 20 h, analyzing (*R*)-(-)-1-octen-3-ol biosynthesis based on time and temperature of incubation was done. In the process, other overnight cultivated cells were respectively inoculated into 100 ml of SC induction medium containing 3 mM linoleic acid and 0.2% Tween-20, followed by an incubation at 15°C and 30°C for 12, 24, 36, and 48 h.

Using the bead beater that consist of sodium phosphate lysis buffer (50 mM sodium phosphate, 1 mM phenylmethanesulfonyl fluoride, 5% glycerol and 2% Triton X-100; pH 6.5) and acid-washed glass beads (0.4–0.6 mm size), the harvested cells were disrupted. The cell lysates were collected by centrifugation at 4°C for 20 min and extracted with diethyl ether (1:1, v/v). And the amount of biosynthesized (*R*)-(-)-1-octen-3-ol was analyzed to compare with the 1-octen-3-ol standard (Sigma) by using Gas chromatography-mass spectrometry (Agilent 789OB GC & 5977B MSD). With programmed column temperature (initial oven temperature of 40°C increased to 120°C at 2°C/min and from 120°C to 240°C at 20°C/min, followed by a holding temperature of 240°C for 5 min), DB-WAX column (60 m × 250 μm × 0.25 μm) and helium carrier gas (flow rate of 1 ml/min) were used for the analysis. The injector temperature was set to 250°C for a total running time of 53 min.

## Results

### Cloning the Genes Encoding Lipoxygenase-1, Lipoxygenase-2, Lipoxygenase-3 and Hydroperoxide Lyase

pGEM Easy T-vectors (Promega), pYES3/CT and pYES2/CT vectors (Invitrogen) were used for cloning each gene. DNA sequencing of each of the genes inserted into the vectors confirmed a perfect 100% homology compared with the cDNA sequences of *T. matsutake* analyzed. The lengths of cDNA of lipoxygenase-1, -2, -3 and hydroperoxide lyase were 3,159 bases, 3,330 bases, 3,855 bases and 1,560 bases, respectively. These translates to protein sequences of 1,052 amino acids, 1,110 amino acids, 1,284 amino acids and 519 amino acids, respectively. Furthermore, molecular weights of recombinant proteins are expected to be approximately 122, 130, 150, and 63 kDa, respectively.

For expression of each gene in yeast, the lipoxygenase-1, -2, -3 genes were inserted in the pYES3/CT vector and the hydroperoxide lyase gene was inserted in the pYES2/CT, followed by transformation of recombinant DNAs into *S. cerevisiae*. The bands of each amplified gene by colony PCR were confirmed to match to the lenght of each gene resolved by electrophoresis on an agarose gel.

### Expression of the Recombinant Protein from Transformants with Each Gene

**Fig. 2:**
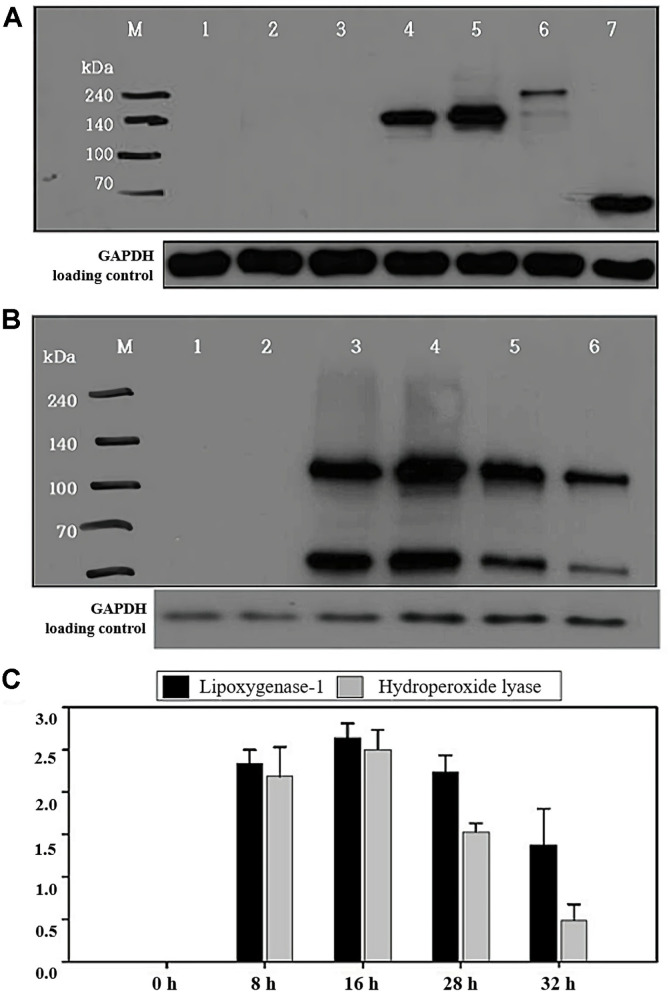
Western blot analysis of lipoxygenase-1, lipoxygenase- 2, lipoxygenase-3 and hydroperoxide lyase proteins. (**A**) The reaction of antibody was conducted. Lane M is the prestained Protein Marker (ELPIS), lane 1 is crud protein of INVSc1 without vector, lane 2 is pYES3/CT without inserted gene, lane 3 is pYES2/CT without inserted gene, lane 4 is lipoxygenase-1, lane 5 is lipoxygenase-2, lane 6 is lipoxygenase-3 and lane 7 is hydroperoxide lyase. (**B**) Western blot analysis of lipoxygenase-1 and hydroperoxide lyase proteins from the co-transformants. Lane M is the pre-stained Protein Marker (ELPIS), lane 1 is crud protein of INVSc1 without vector, lane 2 is lipoxygenase-1 and hydroperoxide lyase from cotransformants induced for 0 h, lane 3 is proteins from cotransformants induced for 8 h, lane 4 is proteins from cotransformants induced for 16 h, lane 5 is proteins from cotransformants induced for 28 h, lane 6 is proteins from cotransformants induced for 32 h. (**C**) Values are ratios of band density to band density of GAPDH at each condition and are mean ± SD.

To analyze the expression of recombinant proteins, transformants that include the genes of lipoxygenase-1, lipoxygenase-2, lipoxygenase-3 and hydroperoxide lyase were incubated respectively in the appropriate SC induction medium with 2% galactose at 30°C for 8 h. After incubation, the induced proteins were analyzed by western blot with an Anti-V5 Mouse monoclonal antibody. The signal of the recombinant proteins encoded by each gene were confirmed to match to the expected molecular weight and lipoxygenase-2 showed the highest level while lipoxygenase-3 showed the lowest level (Fig. 2A).

### Determination of the Gene Set Capable of Synthesizing the Highest amount of (*R*)-(-)- 1-Octen-3-ol

To analyze the production of (*R*)-(-)-1-octen-3-ol based on protein combinations that react with linoleic acid, various combinations of protein extractions were mixed overnight with 1.5 mM linoleic acid at 4°C in a total reaction volume of 3 ml. Then the reactants were analyzed using gas chromatography-mass spectrometry. The results showed that lipoxygenase-1, -2, -3 and hydroperoxide lyase expressed in transformants had specific functions and activity. Furthermore, a distinct difference was observed in the amount of (*R*)-(-)-1-octen-3-ol produced from all protein combinations that reacted with linoleic acid (Table 2). Among the results of (*R*)-(-)-1-octen-3-ol concentration, the set of lipoxygenase-1 and hydroperoxide lyase showed the highest efficiency in generating (*R*)-(-)-1-octen-3-ol.

### Expression of the Recombinant Proteins from the Transformants with Lipoxygenase-1 and Hydroperoxide Lyase Genes

In order to biosynthesize the (*R*)-(-)-1-octen-3-ol in yeast, lipoxygenase-1 and hydroperoxide lyase genes were co-transformed into *S. cerevisiae* competent cells and the expression of recombinant proteins were analyzed by western blot. With the colony PCR, target genes from the co-transformant were amplified and analyzed by electrophoresis with 0.7% agarose gel. As a result, the lipoxygenase-1 and hydroperoxide lyase genes from co-transformants were confirmed to correspond to the size of each gene (Fig. 2B). The co-transformants were incubated in SC induction medium without tryptophan and uracil containing 2% galactose at 30°C for 0, 8, 16, 28, and 32 h. Then the induced proteins were analyzed by western blot and the signal of recombinant proteins encoded by each gene was confirmed to correspond to the expected molecular weight. Lipoxygenase-1 and hydroperoxide lyase from the co-transformants were expressed at higher levels in 16 h, were expressed at higher levels than the other incubation times and the signals of lipoxygenase-1 and hydroperoxide lyase decreased after 28 h of incubation (Fig. 2C).

### Identification of the (*R*)-(-)-1-Octen-3-ol Biosynthesis in Transformants with the Lipoxygense-1 and Hydroperoxide Lyase Genes

To identify the (*R*)-(-)-1-octen-3-ol biosynthesis in transformants with the lipoxygenase-1 and hydroperoxide lyase genes, co-transformants were incubated with 1.5 mM linoleic acid and detection of (*R*)-(-)-1-octen-3-ol biosynthesis was analyzed by gas chromatography-mass spectrometry.

**Fig. 3:**
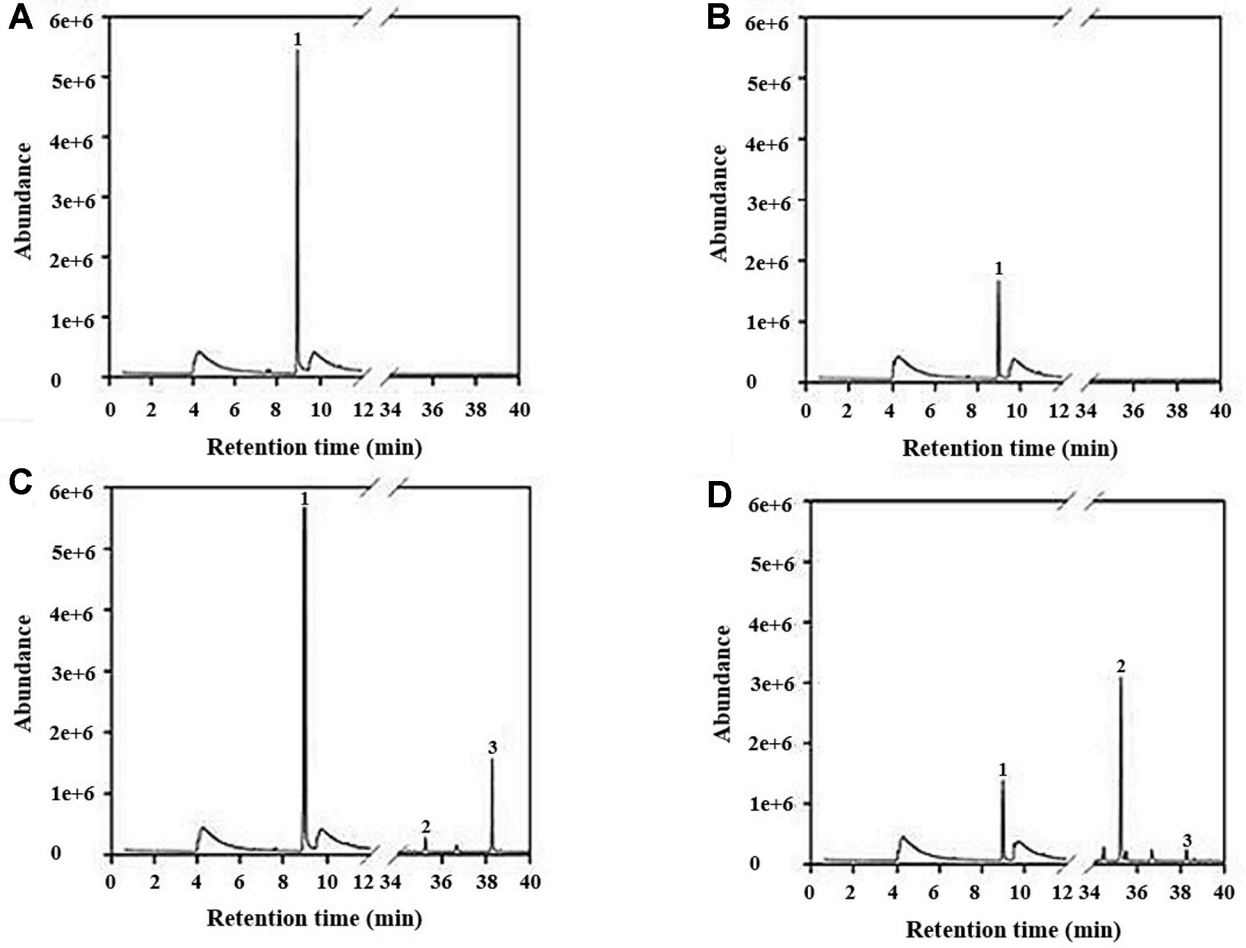
GC-MS chromatograms of bioconversion in transformants with lipoxygenase-1 and hydroperoxide lyase. (**A**) cell lysates incubated without linoleic acid, (**B**) incubated supernatant without linoleic acid, (**C**) cell lysates incubated with linoleic acid, (**D**) incubated supernatant with linoleic acid. 1: ethyl alcohol, 2: 1, 3, 5-undecatriene 3: 1-octen-3-ol.

1-octen-3-ol was detected at retention time of 38.27 min in cell lysates and medium incubated with linoleic acid and the detection showed the peak consistent with the mass spectrum of standard 1-octen-3-ol. The peak comprised 16% of the total area in cell lysates incubated with linoleic acid and 0.8% of the total area in the supernatant incubated with linoleic acid. Therefore, it was identified that transformants with the lipoxygense-1 and hydroperoxide lyase genes are able to convert from linoleic acid to (*R*)-(-)-1-octen-3-ol. Biosynthesis of (*R*)-(-)-1-octen-3-ol from cells showed more efficiency than from the supernatant (Fig. 3).

### Determination of (*R*)-(-)-1-Octen-3-ol Biosynthesis Condition based on Linoleic Acid Concentrations, Time and Temperature of Incubation

To optimize the (*R*)-(-)-1-octen-3-ol biosynthesis process in yeast with the lipoxygenase-1 and hydroperoxide lyase genes, co-transformants were incubated under different conditions. The amount of (*R*)-(-)-1-octen-3-ol biosynthesis was analyzed by gas chromatography-mass spectrometry compared with the 1-octen-3-ol standard (Sigma).

**Fig. 4:**
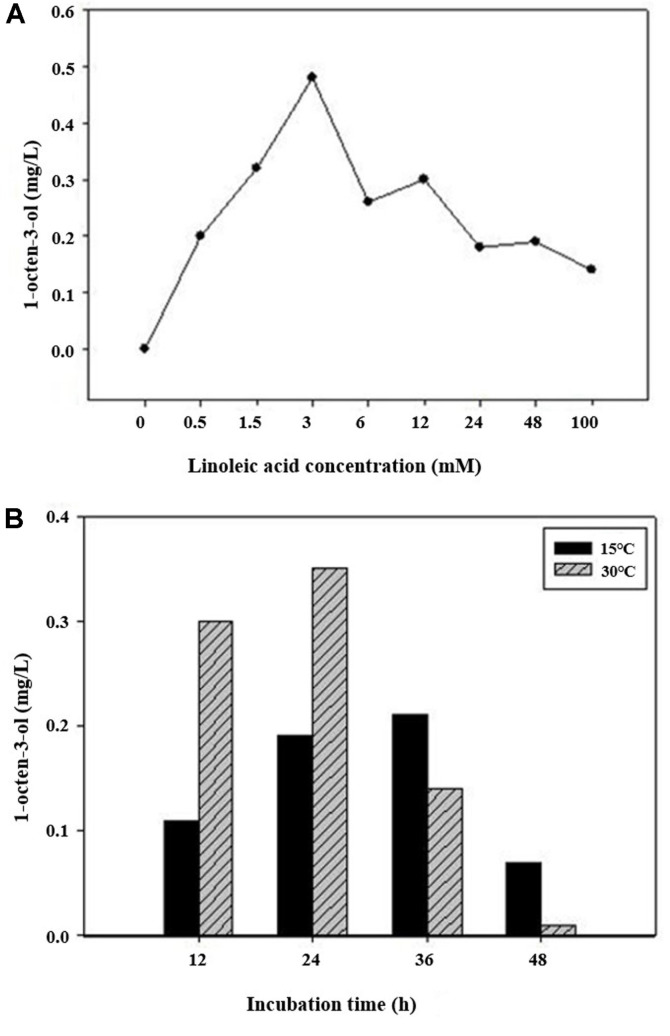
(R)-(-)-1-octen-3-ol biosynthesis in transgenic yeast with lipoxygense-1 and hydroperoxide lyase genes. (**A**) Effect of linoleic acid concentration on (R)-(-)-1-octen-3-ol biosynthesis at 30°C for 20 h, (**B**) effect of incubation time and temperature on (R)-(-)-1-octen-3-ol biosynthesis at 15°C and 30°C for 12, 24, 36 and 48 h.

The biosynthesis of (*R*)-(-)-1-octen-3-ol was increased within a 0–3 mM of linoleic acid range, while a decrease of (*R*)-(-)-1-octen-3-ol biosynthesis was observed. Specially, the amount of (*R*)-(-)-1-octen-3-ol biosynthesis showed the highest degree when co-transformant was incubated with 3mM of linoleic acid. Also the amount of (*R*)-(-)-1-octen-3-ol biosynthesis showed the highest degree at the 30°C incubation comparing with 15°C incubation and the amount of (*R*)-(-)-1-octen-3-ol biosynthesis decreased after 24 h of incubation (Fig. 4).

The optimal condition for (*R*)-(-)-1-octen-3-ol biosynthesis in transgenic yeast was determined to be at a substrate concentration of 3 mM linoleic acid, incubation temperature of 30°C and an incubation time of 24 h.

## Discussion

*Tricholoma matsutake* has a unique flavor and excellent taste and is treated as a high-value food. However, its artificial cultivation is unfortunately hardly possible. Since Hiroshima Forestry Examination Center had artificially cultivated *T. matsutake* by infection in 1983, over 101 studies had been published but none of them have been proven successful in its artificial cultivation. Recently, the National Forest Research Institute, Korea and Taki Chemical Industry, Japan have somewhat succeeded in the artificial cultivation of *T. matsutake* in 2010 and 2018, respectively [17, 20]. Although considerable progressed have been achieved, there are still lots of problems to practically and efficiently cultivate artificially *T. matsutake* [23].

Rather than cultivating the whole mycelium of *T. matsutake*, synthesis of specific extract specially the flavor has been another approach to the problem. Many studies on the flavor of *T. matsutake* have been reported and in 2019, study about flavor fingerprint of *T. matsutake* has been published by National Engineering Research Center of Seafood, China [22]. But in their investigation, components of 3-octanone, 3-octano, 1-octen-3-one, 1-octanol, methanol and 1-pentanol became subject without analysis of gene sequencing information.

Here, we use another approach that centers on the genes responsible for the biosynthesis of 1-octen-3-ol. 1-octen-3-ol is a major flavor component among the none flavor components of *T. matsutake* [25]. Although 1-octen-3-ol is synthesized in other mushrooms or plants, synthesis of 1-octen-3-ol in *T. matsutake* showed a better efficiency [26, 32]. As lipoxygenase and hydroperoxide lyase participate in synthesizing 1-octen-3-ol, those two genes were selected for recombinant. Research of lipoxygenase and hydroperoxide lyase had been published. Dr Ding’s research group analyzed the protein sequence of hydroperoxide lyase from tobacco with 497 amino acids in 2019 [7]. Also Dr Tiwari’s research group analyzed the protein sequence of lipoxygenase from *Eleusine coracana* with 887 amino acids in 2016 [37]. In our study, the cDNA of lipoxygenase-1, -2, -3 and hydroperoxide lyase were translated to protein sequences of 1,052 amino acids, 1,110 amino acids, 1,284 amino acids and 519 amino acids, respectively. Also *E. coli* and *S. cerevisiae* were used for stable and efficient expression of recombinant protein from transformants with lipoxygenase and hydroperoxide lyase. With gene sequencing information about lipoxygenase and hydroperoxide lyase, lipoxygenase-1, lipoxygenase-2, lipoxygenase-3 and hydroperoxide lyase genes were synthesized from cDNA of *T. matsutake* and these genes were cloned into yeast expression vectors. The vectors from *E. coli* were then expressed successfully by the *S. cerevisiae*.

To investigate the efficiency of recombinant protein expression, various combinations of proteins encoded by each gene was mixed overnight with 1.5 mM linoleic acid at 4°C in a total reaction volume of 3 ml. The reactants were then analyzed using gas chromatography-mass spectrometry. Lipoxygenase-1, -2, -3 and hydroperoxide lyase gene expressed in the transformants showed specific functions and activity. In analysis of the protein activity and stability of genes in yeast, the combination of lipoxygenase-1 and hydroperoxide lyase showed the highest efficiency in generating (*R*)-(-)-1-octen-3-ol.

Taken all together, introducing the set of lipoxygenase-1 and hydroperoxide genes into *S. cerevisiae* showed so far the best path to biosynthesize the flavor of *T. matsutake* in yeast. In order to achieve the highest efficiency of synthesis of lipoxygenase-1 and hydroperoxide lyase, co-transformants were incubated in SC induction medium without tryptophan and uracil containing 2% galactose at 30°C for several hours. As results, expression of lipoxygenase-1 and hydroperoxide lyase was higher at 16 h than any other incubation time. The signals of lipoxygenase-1 and hydroperoxide lyase markedly decreased after 16 h. Having identified the best settings for recombinant expression of lipoxygenase-1 and hydroperoxide allow peak biosynthesis production of (*R*)-(-)-1-octen-3-ol. The analyzing (*R*)-(-)-1-octen-3-ol biosynthesis was proceeded according to whether incubation of cell lysates of supernatant and linoleic acid. By the peak of 1-octen-3-ol, the result was analyzed using gas chromatography-mass spectrometry. Without linoleic acid, (*R*)-(-)-1-octen-3-ol was hardly detected in both incubation of cell lysates and supernatant. In addition of linoleic acid, (*R*)-(-)-1-octen-3-ol was detected at a retention time of 38.27 min but biosynthesizing (*R*)-(-)-1-octen-3-ol in cell lysates was better than in the supernatant. It is obvious that incubation in cell lysates with linoleic acid is the most efficient way for (*R*)-(-)-1-octen-3-ol biosynthesis. Also, to determine optimum condition for (*R*)-(-)-1-octen-3-ol biosynthesis, sets of linoleic acid concentration, incubation time and incubation temperature were analyzed separately. The yield of (*R*)-(-)-1-octen-3-ol biosynthesis was optimized with 3 mM of linoleic acid, 24 h of incubation time and 30°C of incubation temperature.

In summary, in this manuscript we investigated the optimal conditions for (*R*)-(-)-1-octen-3-ol biosynthesis. We report that the synthesis with the highest yield was achieved with the set of lipoxygenase-1 and hydroperoxide lyase contained in the cell lysate, 3 mM of linoleic acid, 24 h of incubation time and 30°C of incubation temperature. Large scale production of (*R*)-(-)-1-octen-3-ol is needed but require additional studies in order to efficiently scale up the process.

## Figures and Tables

**Table 1:** Sequences of PCR primers and restriction enzymes.

Primer name		Sequences (5' - 3')	Restriction enzyme
Lipoxygenase-1	Forward	AAGCTTAACACAATGTCCTTAAGCAAGTTTCCG	HindIII
	Reverse	GGTACCACCTTCGTTACATCATACTGTAT	KpnI
Lipoxygenase-2	Forward	GGTACCAACACAATGTTGACGCGGTTATTTAAG	KpnI
	Reverse	GCGGCCGCATATCGAACTGCACAACGAGGG	NotI
Lipoxygenase-3	Forward	AAGCTTAACACAATGTCGATTGATTCTGTTCCA	HindIII
	Reverse	GGTACCATGGCACAGTACTCCCGTTGCCA	KpnI
Hydroperoxide lyase	Forward	GGTACCAACACAATGTCCCTCAAGCATTCTTCC	KpnI
	Reverse	GAATTCTGGATGTTGTGTCCGTGGCGATA	EcoRI

Lipoxygenase and hydroperoxide lyase are amplified for cloning from *Tricholoma matsutake*. Each reactions of amplification were done with forward primer and reverse primers.

**Table 2:** Production of (R)-(-)-1-octen-3-ol based on protein combination.

Protein combination	Retention time (min)	Concentration (mg/L)
Lipoxygenase-1 &	38.27	0.66
Hydroperoxide lyase		
Lipoxygenase-2 &	38.269	0.33
Hydroperoxide lyase		
Lipoxygenase-3 &	38.27	0.58
Hydroperoxide lyase		
Lipoxygenase-1+2 &	38.271	0.42
Hydroperoxide lyase		
Lipoxygenase-1+3 &	38.271	0.38
Hydroperoxide lyase		
Lipoxygenase-2+3 &	38.27	0.27
Hydroperoxide lyase		
Lipoxygenase-1+2+3 &	38.267	0.56
Hydroperoxide lyase		

Production yields of (R)-(-)-1-octen-3-ol in different settings. The combination of lipoxygenase-1 and hydroperoxide lyase showed the highest efficiency in production of (R)-(-)-1-octen-3-ol.

## References

[1] ApSimon J (2009). The total synthesis of natural products.

[2] Assaf S, Hadar Y, Dosoretz CG (1997). 1-Octen-3-ol and 13hydroperoxylinoleate are products of distinct pathways in the oxidative breakdown of linoleic acid by *Pleurotus pulmonarius*. Enzyme Microb. Technol..

[3] Byers JA, Zhang QH, Birgersson G (2004). Avoidance of nonhost plants by a bark beetle, Pityogenes bidentatus, in a forest of odors. Naturwissenschaften.

[4] Byeon SE, Lee J, Lee E, Lee SY, Hong EK, Kim YE (2009). Functional activation of macrophages, monocytes and splenic lymphocytes by polysaccharide fraction from *Tricholoma matsutake*. Arch. Pharm. Res..

[5] Cronin DA, Ward MK (1971). The characterization of some mushroom volatiles. J. Sci. Food Agric..

[6] Da Silva NA, Srikrishnan S (2012). Introduction and expression of genes for metabolic engineering applications in *Saccharomyces cerevisiae*. FEMS Yeast Res..

[7] Ding C, Lu Y, Song Y, Jia R, Jin W, Guo H (2019). Cloning and expression analysis of hydroperoxide lyase gene in *Nicotiana tabacum*. Life Sci. J..

[8] Ding X, Tang J, Cao M, Guo CX, Zhang X, Zhong J (2010). Structure elucidation and antioxidant activity of a novel polysaccharide isolated from *Tricholoma matsutake*. Int. J. Biol. Macromol..

[9] Ebina T (2002). Antitumor effect of a peptide-glucan preparation extracted from a mycelium of *Tricholoma matsutake* (S. I to and Imai) Sing. Biotherapy.

[10] Francotte ER, Richert P (1997). Applications of simulated moving-bed chromatography to the separation of the enantiomers of chiral drugs. J. Chromatogr. A.

[11] Gong M, Su L, Chen Y, Wang F, Cao J (2001). A study on development of Shiro and productive potentialities of *Tricholoma matsutake*. For. Res..

[12] Guerin-Laguette A, Vaario LM, Gill WM, Lapeyrie F, Matsushita N, Suzuki K (2000). Rapid in vitro ectomycorrhizal infection on *Pinus densiflora* roots by *Tricholoma matsutake*. Mycoscience.

[13] Hudault S, Guignot J, Servin AL (2001). *Escherichia coli* strains colonising the gastrointestinal tract protect germfree mice against *Salmonella* typhimurium infection. Gut.

[14] Hur, YH, Kim OK (1991). Studies on the mineral content of edible mushrooms. J. Microbiol. Biotechnol..

[15] Husson F, Bompas D, Kermasha S, Belin JM (2001). Biogeneration of 1-octen-3-ol by lipoxygenase and hydroperoxide lyase activities of *Agaricus bisporus*. Process Biochem..

[16] Ka KH, Hur TC, Park H, Kim HS, Bak WC (2010). Mycelial growth and fairy-ring formation of *Tricholoma matsutake* from matsutake-infected pine trees. J. Korean. Microbiol..

[17] Kataoka R, Siddiqui ZA, Kikuchi J, Ando M, Sriwati R, Nozaki A, Futai K (2012). Detecting nonculturable bacteria in the active mycorrhizal zone of the pine mushroom *Tricholoma matsutake*. J. Microbiol..

[18] Kato H, Matsuda F, Yamada R, Nagata K, Shirai T, Hasunuma T (2013). Cocktail δ-integration of xylose assimilation genes for efficient ethanol production from xylose in *Saccharomyces cerevisiae*. J. Biosci. Bioeng..

[19] Kim JY, Byeon SE, Lee YG, Lee JY, Park J, Hong EK (2008). Immunositimulatory activities of polysaccharides from liquid culture of pine-mushroom *Tricholoma matsutake*. J. Microbiol. Biotechnol..

[20] Kim SS, Lee JS, Cho JY, Kim YE, Hong EK (2010). Effects of C/N ratio and trace elements on mycelial growth and exopolysaccharide production of *Tricholoma matsutake*. Biotechnol. Bioprocess Eng..

[21] Koo CD (2005). Morphological characteristics of *Tricholoma matsutake* ectomycorrhiza. J. Korean Soc. For. Sci..

[22] Li M, Yang R, Zhang H, Wang S, Chen D, Lin S (2019). Development of a flavor fingerprint by HS-GC-IMS with PCA for volatile compounds of *Tricholoma matsutake* Singer. Food Chem..

[23] Lian C, Narimatsu M, Nara K, Hogetsu T (2006). *Tricholoma matsutake* in a natural *Pinus densiflora* forest: correspondence between above-and below-ground genets, association with multiple host trees and alteration of existing ectomycorrhizal communities. New Phytol..

[24] Lynd LR, Van Zyl WH, McBride JE, Laser M (2005). Consolidated bioprocessing of cellulosic biomass: an update. Curr. Opin. Biotechnol..

[25] Matsui K, Sasahara S, Akakabe Y, Kajiwara T (2003). Linoleic acid 10-hydroperoxide as an intermediate during formation of 1-octen-3-ol from linoleic acid in *Lentinus decadetes*. Biosci. Biotechnol. Biochem..

[26] Mau JL, Beelman Rob, Ziegler Grr (1992). 1-Octen-3-ol in the cultivated mushroom, *Agaricus bisporus*. J. Food Sci..

[27] Mosandl A, Heusinger G, Gessner M (1986). Analytical and sensory differentiation of 1-octen-3-ol enantiomers. J. Agric. Food Chem..

[28] Min B, Yoon H, Park J, Oh YL, Kong WS, Kim JG (2020). Unusual genome expansion and transcription suppression in ectomycorrhizal *Tricholoma matsutake* by insertions of transposable elements. PLoS One.

[29] Minami H, K im J S, I kezawa N, T akemura T, K atayama T, Kumagai H (2008). Microbial production of plant benzylisoquinoline alkaloids. Proc. Natl. Acad. Sci. USA.

[30] Murahashi S (1938). The odor of matsutake (Armillaria matsutake Ito er Imai) II. Sci. Pap. I nsr. Phys. Chem. Res..

[31] Park H, Hur TC, Hong YP, Ka KH, Bak WC, Yeo UH (2004). Occurrence of fruiting body of *Tricholoma matsutake* at a Pinus rigida stand in Korea. J. Korean For. Soc..

[32] Pérez AG, Sanz C, Olías R, Olías JM (1999). Lipoxygenase and hydroperoxide lyase activities in ripening strawberry fruits. J. Agric. Food Chem..

[33] Sawahata T, Shimano S, Suzuki M (2008). *Tricholoma matsutake* 1-Octen-3-ol and methyl cinnamate repel mycophagous *Proisotoma minuta* (Collembola: Insecta). Mycorrhiza.

[34] Salas JJ, Sánchez C, García-González DL, Aparicio R (2005). Impact of the suppression of lipoxygenase and hydroperoxide lyase o the quality of the green odor in green leaves. J. Agric. Food Chem..

[35] Sikorski RS, Hieter P (1989). A system of shuttle vectors and yeast host strains designed for efficient manipulation of DNA in *Saccharomyces cerevisiae*. Genetics.

[36] Tenaillon O, Skurnik D, Picard B, Denamur E (2010). The population genetics of commensal *Escherichia coli*. Nat. Rev. Microbiol..

[37] Tiwari A, Avashthi H, Jha R, Srivastava A, Kumar Garg V, Wasudev Ramteke P (2016). Insights using the molecular model of Lipoxygenase from Finger millet (*Eleusine coracana* (L.)). Bioinformation.

[38] Wurzenberger M, Grosch W (1984). The formation of 1octen-3-ol from the 10-hydroperoxide isomer of linoleic acid by a hydroperoxide lyase in mushrooms (Psalliota bispora). Biochim. Biophys. Acta (BBA)-Lipids and Lipid Metabolism.

